# Secondary Asphyxiating Thoracic Dysplasia Due to Multiple Chondromas: A Novel Surgical Report

**DOI:** 10.1093/icvts/ivaf191

**Published:** 2025-08-18

**Authors:** Wenlin Wang, Rajkamal Vishnu, Weiguang Long, Yang Liu

**Affiliations:** Department of Chest Wall Surgery, Guangdong Second Provincial People’s Hospital, Guangzhou, 510422, China; Department of Chest Wall Surgery, Guangdong Second Provincial People’s Hospital, Guangzhou, 510422, China; Department of Cardiothoracic Surgery, Center for Rib Cage Disorders, SRM Institutes for Medical Science, Chennai 600026, India; Department of Chest Wall Surgery, Guangdong Second Provincial People’s Hospital, Guangzhou, 510422, China; Department of Chest Wall Surgery, Guangdong Second Provincial People’s Hospital, Guangzhou, 510422, China

**Keywords:** asphyxiating thoracic dysplasia, bone tumour, chest wall surgery, hypoxia, chest deformity

## Abstract

Asphyxiating thoracic dysplasia (ATD), also known as Jeune syndrome, is a rare and serious genetic condition; its incidence in adult populations is even rarer. A 25-year-old male had a 10-year history of chest wall deformity and progressive dyspnoea. A complex chest wall reconstruction, along with the excision of bone tumours, was performed in view of critical hypoxia. Mechanical ventilation was persistently required postoperatively. However, the patient did improve, and eventually proper chest configuration was restored with a special surgical technique. Histopathological analysis demonstrated the presence of multiple osteochondromas of the ribs. To the best of our knowledge, this is the first reported case of secondary ATD caused by osteochondromas of the ribs.

## INTRODUCTION

Asphyxiating thoracic dysplasia (ATD), commonly referred to as Jeune syndrome, represents a significant clinical challenge due to its life-threatening implications and is characterized by a constricted thoracic cavity.^1^ Asphyxiating thoracic dysplasia severely impairs lung development and function, leading to respiratory failure and death before puberty. Rarely, a few patients survive into adulthood.^2^ Several different surgical approaches have been described, and each procedure is individualized. Unfortunately, reports are limited due to the scarcity of documented cases, the lack of follow-up, and the individualized nature of surgical interventions.^3^ Beyond the primary thoracic constriction caused by ATD, the presence of multiple space-occupying bone tumours can further reduce thoracic capacity and intensify breathing difficulties. This case report details the unprecedented presentation of a 25-year-old male with secondary ATD arising from multiple osteochondromas of the ribs, requiring complex correction of the chest deformity and tumour excision. We believe that this is the first documented case of secondary ATD resulting from a chest wall tumour.

## CASE

A 25-year-old man was hospitalized due to a chest wall abnormality, first noticed 10 years previously without any apparent cause, and progressive difficulty breathing over the past 2 years. Initially, there were no noticeable symptoms, but the chest wall depression gradually worsened. Breathing difficulties began 2 years ago and steadily increased in severity. The condition deteriorated significantly 2 months before admission, necessitating oxygen therapy to alleviate the symptoms. Seeking treatment for his condition, the patient ultimately presented to our medical facility.

**Figure 1.**
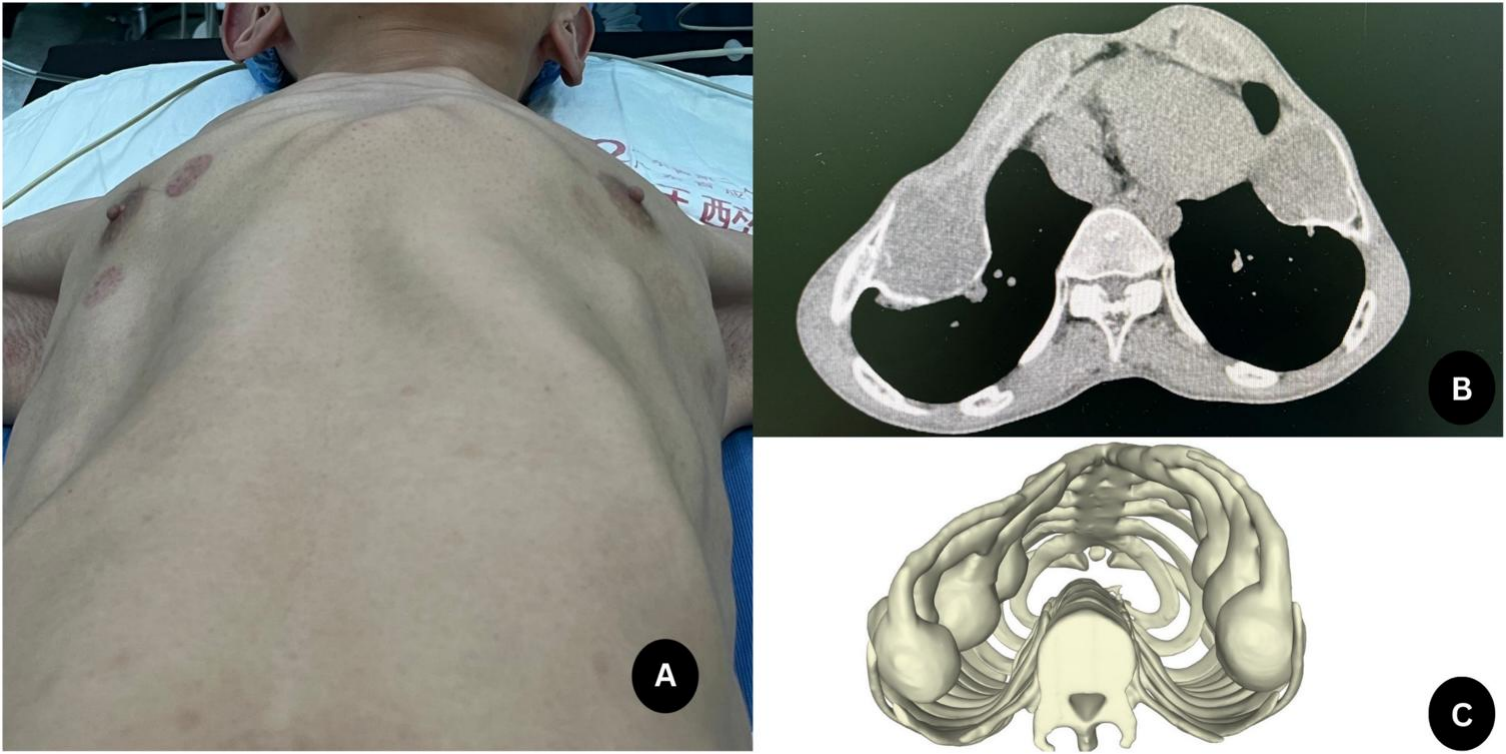
Appearance of the Chest Wall and Computed Tomography Images of the Patient. Preoperative chest wall appearance (A); preoperative chest computed tomography image (B); and 3-dimensional reconstruction image (C).

On physical examination, the anterior chest wall at the centre and costal arch was slightly lordotic and depressed on both sides of the anterior chest wall. (**Figure 1A**). Breath sounds in both lungs were diminished, no rales were heard, and oxygen saturation was 70% with oxygen support. Computed tomography imaging showed severe anterior chest wall deformity, suggestive of ATD (**Figure 1B**). The intercostal spaces were crowded, with multiple mass lesions arising from the costochondral joints of the sixth to ninth ribs bilaterally (**Figure 1C**). The mass protruded into the thorax, significantly narrowing the chest cavity, as did the anterior chest wall deformity. The patient was diagnosed with secondary ATD caused by a bone tumour. Emergency surgery was warranted because the patient was deteriorating.

**Figure 2.**
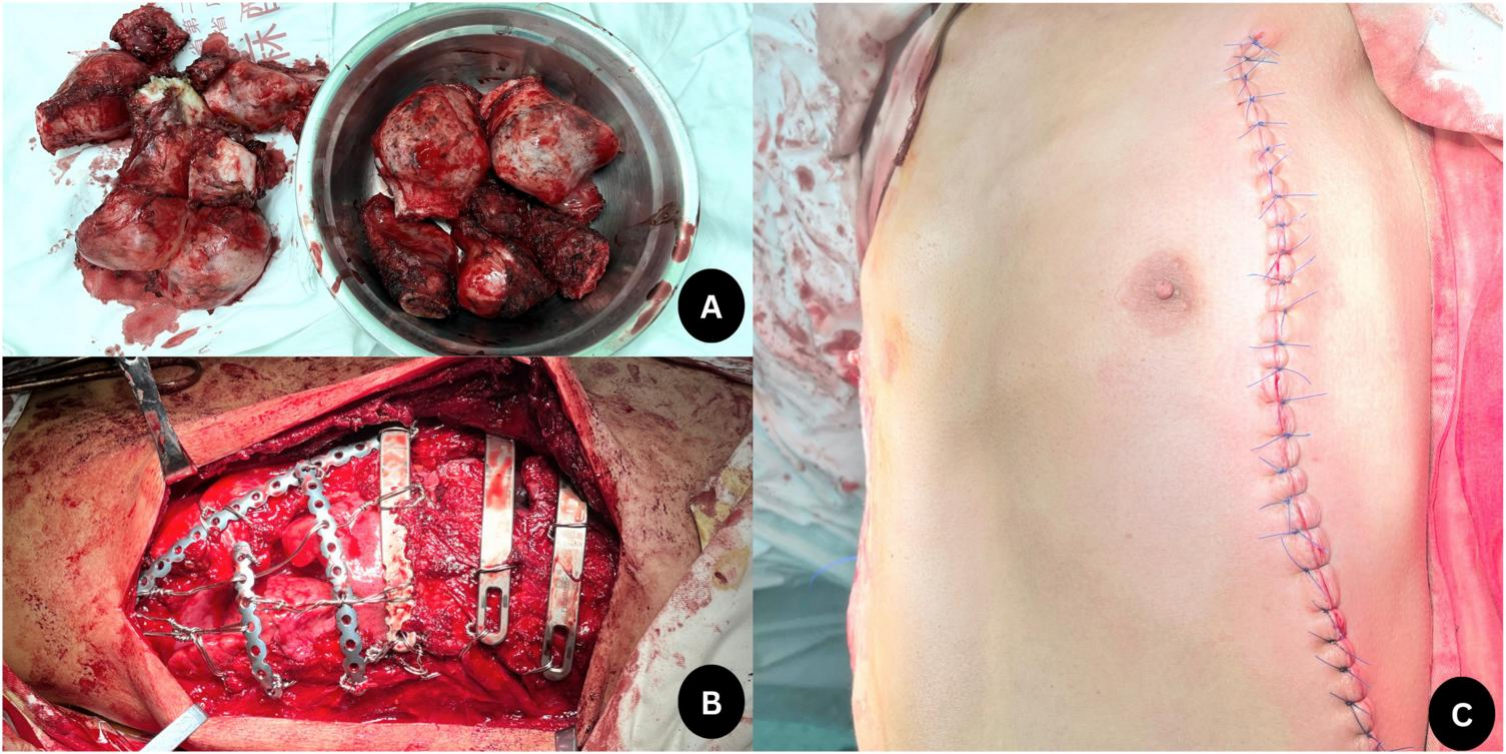
Perioperative and Postoperative Pictures. Multiple bar placement (A); resected specimen (B); and postoperative appearance of the chest wall (C).

With the patient under general anaesthesia and in the supine position, a vertical skin incision was made on the lateral aspect of the chest, corresponding to the anterior axillary line. A bulky tumour mass involving the sixth to ninth ribs was excised with adequate margins on both sides. The residual rib defect was reconstructed using MatrixRIB plates (DePuy Synthes/Johnson & Johnson, Brunswick, NJ, USA) and stainless steel wires (**Figure 2A**). The carinated part was repaired using 3 pectus steel bars. These arc-shaped bars were strategically placed above the sternum and aligned before the lateral depressions. The ribs and costal cartilages at the depression were lifted and fixed to the bars using steel wires, eliminating the depressions (Wang Procedure). Haemostasis was achieved, and drain tubes were placed. The operative time was 264 min, and intraoperative blood loss was 950 ml. The wound was closed in layers. The final histopathology report revealed an osteochondroma (costochondroma) of the rib with negative margins (**Figure 2B and C**).

Postoperatively, the patient required prolonged mechanical ventilatory support. On day 7, a tracheostomy was performed. Over time, his respiratory function improved, and he was gradually weaned off ventilatory support, leading to tracheostomy closure after adequate physiotherapy. The patient demonstrated no signs of hypoxia or respiratory distress. He was discharged and remains under follow-up.

During the first follow-up visit a week after the operation, his tracheostomy site had healed well, but the patient complained of mild chest discomfort during activities. The Visual Analogue Score for pain was 3, which was managed with analgesics. The chest wound was healthy. At 3 months follow-up, the patient had good respiratory excursions, was free of symptoms, and was able to perform routine activities of daily living. The computed tomography scan showed no signs of tumour recurrence and implants in situ with no displacement. Chest wall integrity was maintained (see **Figure S1**).

## DISCUSSION

The prognosis of ATD historically has been grim, with a substantial mortality rate in early childhood, primarily due to respiratory decompensation. However, the recent literature reports a handful of patients with ATD surviving into adulthood with late symptom onset and undergoing successful surgical correction.^4^^,^^5^ Osteochondroma of the rib, although benign, is notorious for complications such as pneumothorax, pleural effusion, haemothorax, diaphragm injury, compression symptoms, and pericardial effusion. It is usually seen during puberty; if the patient experiences any growth in adulthood, the chance of malignant transformation is greater. However, to prevent complications and malignant transformations, an operation is recommended.^6^ Due to the heterogeneity of the disease, respiratory compromise in adults presents a significant challenge, given the underlying lung pathology and additional chest wall compression caused by the defect. This situation necessitates an early and tailored approach to management. Secondary ATD is a newly identified entity that has not been previously reported in the literature. It presents a unique and challenging scenario during surgical management due to secondary lung compression from multiple bone tumours, in addition to the pre-existing narrow chest cavity associated with ATD.

In summary, we successfully reconstructed a rare chest wall disorder involving multiple bone tumours. This approach proved effective in alleviating respiratory distress and restoring thoracic integrity. Our findings contribute to the growing body of knowledge by documenting a novel case of secondary ATD in a 25-year-old patient.

## Supplementary Material

ivaf191_Supplementary_Data

## Data Availability

The data underlying this article are available in the article and in its online supplementary material.
