# Supplementary material for: Secondary Asphyxiating Thoracic Dysplasia Due to Multiple Chondromas: A Novel Surgical Report
Source: Interdiscip Cardiovasc Thorac Surg. 2025 Aug 18;40(9):ivaf191. doi: 10.1093/icvts/ivaf191 (PMC12408469; doi:10.1093/icvts/ivaf191)
Supplement: ivaf191_Supplementary_Data [file ivaf191_supplementary_data.zip › Supplementary Figure legend.docx]

Supplementary Figure legend:

Supplementary Figure 1: Post-operative 3D Reconstruction during follow-up.
