# Supplementary figures and images for: Secondary Asphyxiating Thoracic Dysplasia Due to Multiple Chondromas: A Novel Surgical Report
Source: Interdiscip Cardiovasc Thorac Surg. 2025 Aug 18;40(9):ivaf191. doi: 10.1093/icvts/ivaf191 (PMC12408469; doi:10.1093/icvts/ivaf191)

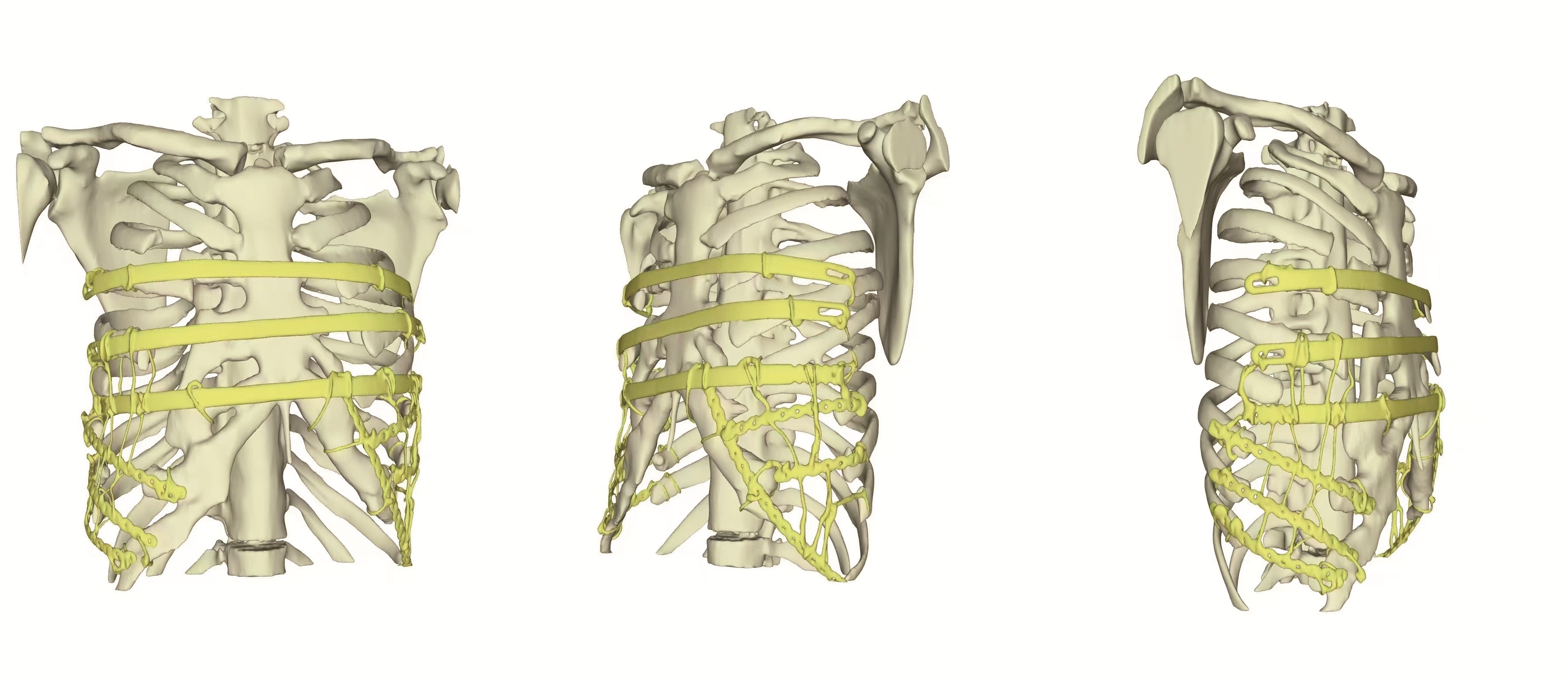

Supplement: ivaf191_Supplementary_Data [file ivaf191_supplementary_data.zip › Supplimentary files.tif]
